# Effects of dietary polyunsaturated fatty acid sources on expression of lipid-related genes in bovine milk somatic cells

**DOI:** 10.1038/s41598-020-71930-x

**Published:** 2020-09-09

**Authors:** Einar Vargas-Bello-Pérez, Nathaly Cancino-Padilla, Carolina Geldsetzer-Mendoza, María Sol Morales, Heidi Leskinen, Philip C. Garnsworthy, Juan J. Loor, Jaime Romero

**Affiliations:** 1 Faculty of Health and Medical Sciences, Department of Veterinary and Animal Sciences, University of Copenhagen, Grønnegårdsvej 3, 1870 Frederiksberg C, Denmark; 2 Departamento de Ciencias Animales, Facultad de Agronomía e Ingeniería Forestal, Pontificia Universidad Católica de Chile, Casilla 306., Santiago, 6904411 Chile; 3 Departamento de Fomento de la Producción Animal, Facultad de Ciencias Veterinarias y Pecuarias, Universidad de Chile, Av. Santa Rosa 11735, La Pintana, Santiago, Chile; 4 Milk Production, Production Systems, Natural Resources Institute Finland (Luke), 31600 Jokioinen, Finland; 5 School of Biosciences, The University of Nottingham, Sutton Bonington Campus, Loughborough, LE12 5RD UK; 6 Department of Animal Sciences and Division of Nutritional Sciences, University of Illinois, Mammalian NutriPhysioGenomics, Urbana, 61801 USA; 7 Laboratorio de Biotecnología en Alimentos, Unidad de Alimentos, Instituto de Nutrición y Tecnología de los Alimentos, Universidad de Chile, Avda. El Líbano 5524, Macul, 7830490 Santiago, Chile

**Keywords:** Animal physiology, Fat metabolism

## Abstract

The objective of this study was to compare the effect of contrasting sources of dietary n-6 and n-3 PUFA on expression of genes related to lipid metabolism in dairy cows. During 63 days, fifteen lactating cows were assigned to a control or basal diet containing no added lipid (n = 5 cows); and treatment diets supplemented with SO (n = 5 cows; unrefined soybean oil; 2.9% of DM) or FO (n = 5 cows; fish oil manufactured from salmon oil; 2.9% of DM). Plasma for fatty acid (FA) analysis and milk somatic cells (MSC) were obtained from all cows at the beginning of the study (day 0) and on days 21, 42 and 63. Plasma was used to determine FA transport dynamics. Compared with control and FO, plasma from SO had increased contents of C18:1 *cis*-9, C18:1 *trans*-11, C18:2 *cis*-9, *trans*-11 and total monounsaturated FA. On the other hand, compared with control and SO, FO increased plasma contents of C20:3 n-3, C20:3 n-6, C20:4 n-6, C20:5 n-3, C22:6 n-3 and total polyunsaturated FA. Moreover, plasma C18:3 n-3 and C20:5 n-3 increased over time for all diets. Compared with control, SO downregulated *ACACA*, *INSIG1*, and *DGAT1*, whereas FO downregulated *ACACA*, *PPARGC1*, *LPIN1* and *FABP3* on day 63, in MSC. At different time-points, SO and FO downregulated genes related to synthesis and intracellular transport of FA, synthesis of triglycerides, and transcription factors.

## Introduction

Polyunsaturated fatty acids (PUFA) have been used in dairy cow diets to increase unsaturated fatty acids (FA) such as C18:1 *cis*-9, C18:1 *trans*-11, and C18:2 *cis*-9, *trans*-11 in dairy products^1,2^. Sources for these FA can be oils of vegetable (e.g., olive, soybean) and marine (e.g., salmon oil) origin. When cows are fed dietary oils, different responses take place at molecular levels, such as changes in expression of genes related to lipid metabolism at adipose tissue^3,4^ or at mammary gland^5–7^ levels. In this regard, we have observed that degree of FA saturation can trigger differential effects in expression of lipid-related genes in the mammary gland^6^. In fact, those alterations were observed in different biological functions, such as FA synthesis (*ACACA*), acetate and FA activation and intra-cellular transport (*FABP3*, *FABP4*), lipid droplet formation (*PLIN2*), and transcription regulation (*THRSP*), and this was observed over a period of 63 days of lipid supplementation with either palm oil (as a saturated FA source) or olive oil (as an unsaturated FA source). We have also reported that at in vitro level, responses of mammary gland cells to lipid supplementation will depend on individual FA structure, such as chain length, degree of saturation, and orientation of FA double bonds^8^.

Although soybean oil (SO) and fish oil (FO) can be considered as PUFA sources, the number and location of double bonds seems to exert different effects on transcription of isogenic genes. This difference has been reported in transcription of lipogenic genes in subcutaneous adipose tissue^6^, but not at mammary gland level. Until now, we know^6^ that there are differential effects when comparing dietary saturated FA (i.e., hydrogenated vegetable oil) with unsaturated FA sources (i.e., olive oil). However, it remains unclear if dietary PUFA from different sources will exert differential expression on genes related to lipid metabolism at mammary gland level over a relatively long-term lipid supplementation. Thus, the objective of this study was to compare the effect of contrasting sources of dietary n-6 and n-3 PUFA on expression of genes related to lipid metabolism in dairy cows. For this purpose, we chose SO as a rich source of C18:2 *cis* n-6, and FO as a rich source of C20:5 n-3 and C22:6 n-3 over a relatively long period of supplementation (63 days). For gene expression, milk somatic cells (MSC) were used instead of percutaneous biopsies of the mammary gland. MSC are representative sources of RNA in mammary gland tissue^9^, and they have been successfully used in previous experiments focused on mammary gland transcriptome^6,10,11^.

## Results and discussion

### Animal performance

Overall production performance, milk composition and milk FA profile were reported previously^1^. Briefly, body weight (634 kg), body condition score (2.55), milk production (43 kg/day), milk fat (1.52 kg/day), and milk protein (1.50 kg/day) were not affected by treatments. Saturated fatty acids in milk fat were decreased with SO and FO compared with control. C18:2 *cis* n-6 was increased with SO whereas C18:2 *cis*-9, *trans*-11, C20:3 n-3, C20:3 n-6, C20:5 n-3, and C22:6 n-3 were highest with FO.

### Plasma fatty acid transport

In this study, plasma was used as an indicator of FA transport dynamics which is one of the main factors controlling lipid utilization by tissues and, ultimately, milk FA profiles. As reported in other studies dealing with dietary lipids in dairy cows^12,13^, plasma FA were mostly represented by C16:0, C18:0, C18:1 *cis-*9 and C18:2 *cis* n-6. Effects of treatment, sampling time and their interactions are reported in Table 1. Mean values for plasma FA correspond to means of 0, 21, 42 and 63 days. In this study, compared with control and FO, SO increased proportions of C18:1 *cis-*9, C18:1 *trans-*11, C18:2 *cis-*9, *trans-*11 and total MUFA in plasma. On the other hand, compared with control and SO, FO increased plasma contents of C20:3 n-6, C20:4 n-6, C20:5 n-3, C22:6 n-3 and total PUFA.

**Table 1:** Plasma fatty acid composition from cows fed control, soybean oil (SO), and fish oil (FO) dietary treatments.

Fatty acid (g/100 g of fatty acid)	Diets^1^						
	Control	SO	FO	SEM	Diet (D)	Time (T)	D × T
C10:0	0.53	0.81	0.51	0.20	0.08	0.99	0.62
C14:0	0.79	0.99	0.55	0.33	0.10	0.82	0.34
C15:0	0.60^a^	0.33^b^	0.55^a^	0.19	< 0.001	0.49	0.20
C16:0	11.7	11.3	10.7	1.54	0.13	0.39	0.17
C17:0	0.38	0.36	0.31	0.13	0.27	0.97	0.42
C18:0	16.4	15.1	15.3	1.33	0.41	0.25	0.43
C18:1 *trans-*11	0.57^b^	0.85^a^	0.67^b^	0.15	< 0.001	0.93	0.70
C18:1 *cis-*9	25.2^b^	27.5^a^	21.6^c^	1.47	< 0.001	0.47	0.78
C18:2 *cis* n-6	27.0	28.0	25.0	2.28	0.16	0.06	0.08
C18:2 *trans* n-6	0.70	0.63	0.67	0.13	0.44	0.90	0.65
C18:3 n-3	4.51	4.60	3.38	2.45	0.39	0.03	0.10
C18:3 n-6	2.02	2.38	2.00	0.72	0.72	0.49	< 0.001
C18:2 *cis-*9, *trans-*11	1.34^b^	1.95^a^	1.28^b^	0.51	0.05	0.99	0.03
C20:3 n-3	1.75	1.35	2.11	0.58	0.19	0.44	0.95
C20:3 n-6	2.46^a^	0.84^b^	2.50^a^	0.56	< 0.001	0.89	0.50
C20:4 n-6	1.21^b^	0.74^b^	3.71^a^	0.48	< 0.001	0.42	< 0.001
C20:5 n-3	0.94^b^	0.76^b^	4.96^a^	0.60	< 0.001	0.01	0.01
C22:6 n-3	1.35^b^	1.22^b^	3.72^a^	0.49	< 0.001	0.19	0.28
Others	0.32^b^	0.46^a^	0.35^b^	0.04	0.01	0.84	0.76
Σ Saturated fatty acids	30.3	28.9	27.9	3.26	0.18	0.13	0.98
Σ Monounsaturated fatty acids	26.0^b^	28.1^a^	22.3^c^	3.00	< 0.001	0.49	0.78
Σ Polyunsaturated fatty acids	43.2^b^	42.4^b^	49.3^a^	3.03	< 0.001	0.38	0.62

Values are LSM and pooled SEM, n = 45.

*SO* supplemented with 29 g/kg DM soybean oil, *FO* supplemented with 29 g/kg DM fish oil, *SEM* standard error of the mean.

^a,b,c^Means in the same row with different superscript letters are significantly different (Diet P < 0.05).

^1^Control, no fat supplement.

**Figure 1:**
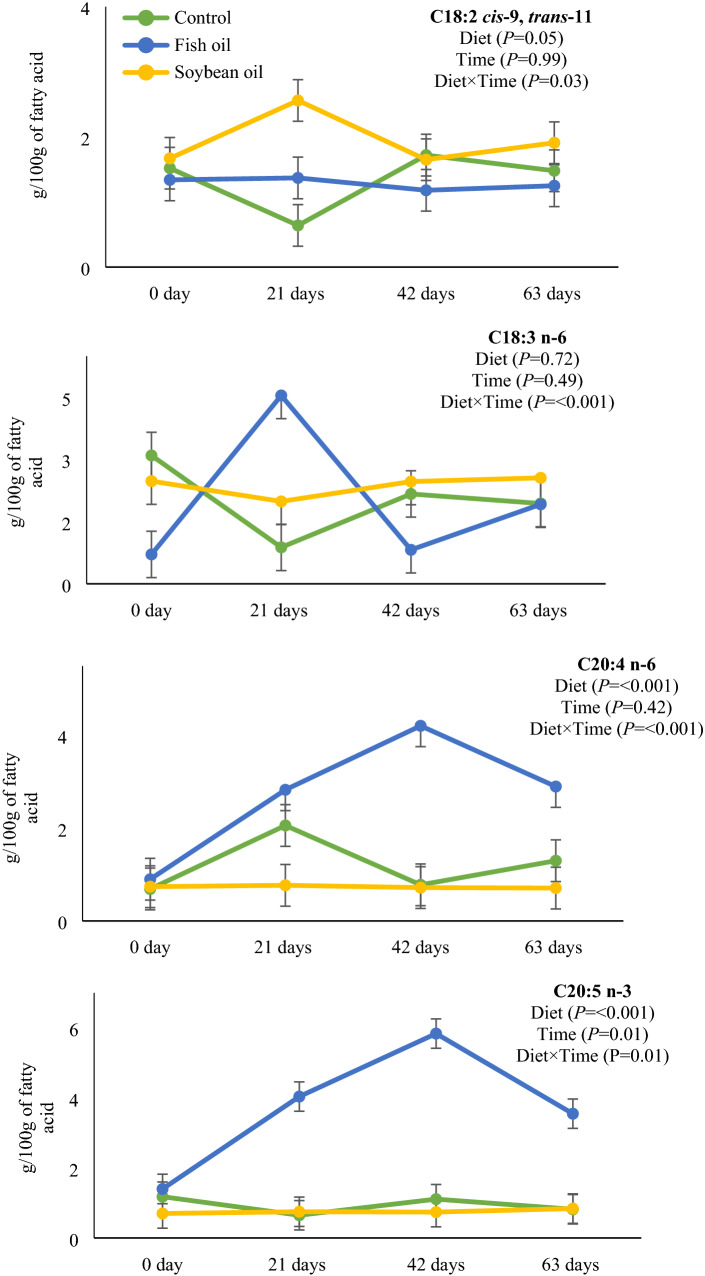
Interactions between treatments (control, soybean oil and fish oil) and experimental periods for plasma fatty acids. Bars denote standard errors of the means. Data are based on 5 cows per treatment.

Moreover, contents (g/100 g) of C18:3 n-3 (from 2.4 at day 0 to 6.5 at day 63) and C20:5 n-3 (from 2.7 at day 0 to 1.7 at day 63) changed over time. C18:2 *cis*-9, *trans*-11, C18:3 n-6, C20:4 n-6 and C20:5 n-3 had time by treatment interactions (Fig. 1). With regard to C18:2 *cis*-9, *trans*-11, the content remained constant over 63 days in FO, whereas in SO it reached a higher content on day 21 and then decreased to its basal content (day 0) on days 42 and 63. C18:3 n-6 content was increased on day 21 by FO, whereas with SO its content remained constant from day 0 to 63. In both C20:4 n-6 and C20:5 n-3 FO increased their contents on days 21 and 42, whereas for SO contents remained constant from day 0 to 63.

The temporal variations observed for C18:2 *cis*-9, *trans*-11, C18:3 n-6, C20:4 n-6 and C20:5 n-3 mirrored the FA profile from dietary treatments. These temporal variations may have also reflected changes in rumen biohydrogenation over time. For example, in a previous study, the higher contents of C18:2 *cis* n-6 in SO diet may have elicited an increase in ruminal biohydrogenation intermediate C18:2 *cis*-9, *trans*-11^12^. In addition, in an earlier study the higher total PUFA in FO resulted in increased contents of several 18-carbon intermediates and long-chain n-3 in plasma compared with hydrogenated palm oil over a 21-d period of supplementation^13^. In this study, perhaps the most important feature from plasma FA results is that dietary lipids provoked the most intense changes in a period of 21 days for C18:2 *cis* n-6 and C18:3 n-3, and for long-chain FA such as C20:4 n-6 and C20:5 n-3, a period of 42 days seemed to result in an upturn. Overall, in this study, FA profile data suggest that FO elicited stronger shifts in rumen biohydrogenation pathways than SO. It is possible that rumen microorganisms took longer to adapt to FO than SO. In order to confirm this, future studies should consider analyzing rumen fluid FA profile and microbiome.

### Genes selected to study fatty acid metabolism

Relative abundance of genes related to lipid metabolism from MSC was obtained, using the control treatment (no lipid supplementation) as a reference condition (Table 2). Of the genes analyzed, those that did not change their expression across sampling periods were selected, with the purpose of relating observed changes with dietary lipid supplementation and not with stage of lactation of animals.

**Table 2:** Relative expression of genes involved in lipid metabolism in milk somatic cells from cows fed control (no fat supplementation) on days 21, 42 and 63 using the relative abundance of the onset of the experiment (day 0; no fat supplementation) as the reference condition.

Gene	Day	Relative abundance	SE	P-value	Regulation
*ACACA*	21	5.059	0.114–589.268	0.188	
	42	5.497	0.075–307.535	0.075	
	63	3.847	0.096–191.216	0.148	
*FADS2*	21	3.784	0.150–55.669	0.088	
	42	3.459	0.100–46.236	0.143	
	63	1.888	0.090–20.405	0.369	
*FASN*	21	48.221	1.649–1,144.705	< 0.001	UP
	42	52.847	3.743–700.673	< 0.001	UP
	63	6.729	0.571–59.637	0.007	UP
*SCD*	21	2.707	0.465–20.204	0.037	UP
	42	1.665	0.129–26.317	0.433	
	63	1.022	0.216–5.514	0.966	
*ADFP*	21	0.404	0.008–6.932	0.297	
	42	0.16	0.007–5.581	0.046	DOWN
	63	1.279	0.016–17.358	0.761	
*INSIG1*	21	4.712	0.091–171.803	0.107	
	42	5.533	0.183–238.627	0.072	
	63	2.313	0.086–55.007	0.321	
*SCAP*	21	2.879	0.077–131.061	0.266	
	42	11.228	0.430–286.384	0.008	UP
	63	1.013	0.095–11.911	0.987	
*SREBF1*	21	3.169	0.232–46.744	0.072	
	42	2.897	0.767–13.521	0.006	UP
	63	1.667	0.381–8.014	0.194	
*THRSP*	21	10.556	0.538–277.759	0.012	UP
	42	11.937	0.164–805.185	0.023	UP
	63	0.922	0.063–17.463	0.911	
*PPARGC1*	21	1.066	0.102–22.011	0.937	
	42	0.524	0.028–9.518	0.404	
	63	1.154	0.076–22.280	0.829	
*DGAT1*	21	2.367	0.047–68.188	0.345	
	42	3.51	0.119–194.295	0.221	
	63	3.984	0.069–87.434	0.127	
*DGAT2*	21	0.109	0.002–2.942	0.023	DOWN
	42	0.647	0.005–10.895	0.641	
	63	0.216	0.005–5.865	0.110	
*LPIN1*	21	2.578	0.074–60.214	0.301	
	42	6.495	0.220–513.932	0.072	
	63	2.593	0.082–52.633	0.242	
*LPL*	21	3.853	0.327–47.144	0.024	UP
	42	5.482	0.305–137.114	0.022	UP
	63	3.781	0.562–30.735	0.016	UP
*FATP*	21	3.684	0.044–321.504	0.253	
	42	67.492	1.871–1,823.015	< 0.001	UP
	63	6.933	0.352–141.529	0.023	UP
*VLDLR*	21	3.572	0.321–34.891	0.053	
	42	3.447	0.379–36.882	0.045	UP
	63	5.69	0.203–142.349	0.040	UP
*ACSL1*	21	3.626	0.746–22.018	0.004	UP
	42	1.876	0.138–27.129	0.315	
	63	4.99	0.409–40.339	0.007	UP
*ACCS2*	21	1.84	0.230–17.743	0.318	
	42	0.223	0.008–3.841	0.050	DOWN
	63	0.845	0.094–9.559	0.787	
*FABP3*	21	1.247	0.130–13.126	0.647	
	42	1.058	0.252–6.559	0.887	
	63	0.496	0.098–4.235	0.134	
*FABP4*	21	2.115	0.376–12.273	0.132	
	42	4.802	1.088–34.271	0.001	UP
	63	4.481	1.285–41.824	< 0.001	UP

### Effects of SO and FO on lipid metabolism-related genes in MSC

Relative abundance of genes involved in lipid metabolism in MSC from SO (Table 3) and FO (Table 4) was examined using day 0 (no fat supplementation) as the reference condition for each sampling time (21, 42, and 63 days). Genes related to the main biological processes of fat metabolism in MSC affected by dietary oil supplementation were synthesis and desaturation of FA (*ACACA*, *FADS2*), intracellular transport of FA (*FABP3*), synthesis of triglycerides (*DGAT1*, *LPIN1*) and transcription factors (*INSIG1*, *PPARGC1*).

**Table 3:** Relative abundance of genes involved in lipid metabolism in milk somatic cells from cows fed soybean oil (SO) using the onset of the experiment (day 0; no fat supplementation) as the reference condition (SO vs. day 0 in SO, in each sampling time).

Gene	Day	Abundance	SE	*p* Value	Regulation
*ACACA*	21	1.466	0.301–8.665	0.355	
	42	0.09	0.016–0.524	< 0.001	DOWN
	63	0.129	0.026–0.737	< 0.001	DOWN
*FASD2*	21	1.100	0.529–2.166	0.583	
	42	1.048	0.533–2.109	0.804	
	63	1.259	0.687–2.355	0.15	
*INSIG1*	21	0.363	0.123–1.282	0.004	DOWN
	42	0.413	0.146–1.155	< 0.001	DOWN
	63	0.201	0.081–0.521	< 0.001	DOWN
*PPARGC1*	21	0.668	0.054–7.272	0.466	
	42	0.534	0.151–2.018	0.084	
	63	0.843	0.103–5.713	0.696	
*DGAT1*	21	0.403	0.219–0.713	< 0.001	DOWN
	42	0.749	0.379–1.752	0.167	
	63	0.542	0.329–0.909	0.001	DOWN
*LPIN1*	21	0.544	0.106–2.757	0.12	
	42	0.53	0.170–1.576	0.035	DOWN
	63	0.738	0.200–2.893	0.384	
*FABP3*	21	1.247	0.130–13.126	0.647	
	42	1.058	0.252–6.559	0.887	
	63	0.496	0.098–4.235	0.134	

*SO* supplemented with 29 g/kg DM soybean oil.

**Table 4:** Relative abundance of genes involved in lipid metabolism in milk somatic cells from cows fed fish oil (FO) using the onset of the experiment (day 0; no fat supplementation) as the reference condition (FO vs. day 0 in FO, in each sampling time).

Gene	Day	Abundance	SE	*p* Value	Regulation
*ACACA*	21	0.086	0.021–0.430	< 0.001	DOWN
	42	0.055	0.011–0.390	< 0.001	DOWN
	63	0.068	0.011–0.351	< 0.001	DOWN
*FASD2*	21	0.686	0.268–2.044	0.124	
	42	0.851	0.154–5.180	0.730	
	63	0.965	0.334–2.866	0.897	
*INSIG1*	21	1.494	0.387–11.075	0.329	
	42	0.851	0.154–5.180	0.730	
	63	0.799	0.184–5.502	0.605	
*PPARGC1*	21	0.314	0.051–1.718	0.009	DOWN
	42	0.262	0.029–2.259	0.015	DOWN
	63	0.221	0.034–1.643	0.004	DOWN
*DGAT1*	21	1.28	0.736–2.150	0.117	
	42	2.089	0.725–27.553	0.105	
	63	1.279	0.798–1.960	0.073	
*LPIN1*	21	0.345	0.061–1.375	0.009	DOWN
	42	0.277	0.043–1.691	0.005	DOWN
	63	0.277	0.058–1.095	0.001	DOWN
*FABP3*	21	0.237	0.042–1.389	0.002	DOWN
	42	0.122	0.013–1.645	0.002	DOWN
	63	0.210	0.048–1.614	0.001	DOWN

*FO* supplemented with 29 g/kg DM fish oil.

In both types of oil supplementation, a downregulation of genes was observed. In SO, genes affected were *ACACA*, *INSIG1*, *DGAT1* and, *LPIN1*, whereas FO affected expression of *ACACA*, *PPARGC1*, *LPIN1* and *FABP3*. Previous studies have reported that consumption of FO may decrease milk fat synthesis (milk fat depression syndrome, MFD), which results from formation of some antilipogenic intermediates of biohydrogenation in the rumen, such as C18:1 *trans*-10^14^. Diet-induced MFD generates a downregulation of genes related to metabolism of FA in the mammary gland^15^, such as *ACACA*, *FASN*, *INSIG1*, *SCD*, *SREBF1*, and *THRSP*^5,16,17^. In the present study, MFD was not observed^1^ and specific biohydrogenation intermediates were not observed in plasma. However, there were changes in expression of genes that have been associated with MFD, especially in cows supplemented with FO. In this study, it is possible that 2.9% DM of supplementation was sufficient to cause mild nutrigenomic changes at the mammary gland level without deleterious effects on milk fat content.

With regard to FA synthesis, both SO and FO downregulated expression of *ACACA*, a regulatory enzyme for synthesis of short chain FA and palmitate^17^. This enzyme is regulated by various factors associated with glucose content of the cell, glucose:glucagon ratio, and blood thyroxine concentration^18,19^. Our results differ from studies where relative expression of *ACACA* was not affected when cows were fed olive oil^6^ and soybean oil^20^ and when goats were supplemented with soybean^21^. The reduction in *ACACA* regulation caused by SO and FO could be related to an increase in dietary PUFA supply, which is normally related to downregulation of transcription regulatory genes such as *SREBF1*. *SREBF1* is responsible for controlling expression of genes related to synthesis of FA^22^, among which are *ACACA* and *INSIG1*.

The lack of treatment effect on activity of *FASD2* was unexpected. *FASD2* is an enzyme responsible for delta-6 desaturase and, therefore, to partly regulating synthesis of further omega-3 and -6 FA from C18:2 *cis* n-6 and C18:3 n-3^19^. With regard to FASD genes, studies conducted in Holstein cows^23^ and women from China^24^ and Bangladesh^25^, have reported that changes in milk concentration of PUFA are related to maternal genetics and diet. However, caution must be exercised when extrapolating from human studies, as bovine lipid metabolism is different from that of humans.

*FABP3* is a protein that allows hydrophobic substances to be transported in an aqueous medium, such as cytoplasm of epithelial cells, and it is responsible for mobilizing long-chain FA^26^. Cows supplemented with SO showed no change in *FABP3* expression, but in cows supplemented with FO, downregulation of *FABP3* occurred from day 21 onwards. It has been reported that an increase in content of palmitic and stearic acids in cell cultures generates an increase in expression of *FABP3*^27^. In the present study, a higher content of palmitic acid in FO diet was observed, but once metabolized in the animals, FO generated a reduction in the content of palmitic acid in milk^1^ compared to the control and SO groups. That could partly explain why changes in *FABP3* expression were more evident in the FO group. Another cause of the decrease in *FABP3* expression in FO, is the decrease in milk short-chain FA (C6:0, C8:0 and C10:0), which was observed in SO and FO^1^. In mammary epithelial cells from goat^28^, supplementation of acetate and butyrate led to an upregulation of *PPARG* and therefore increased expression of *FABP3*, as well as genes related to triacylglycerol accumulation and lipid droplet formation, such as *SCD*, *SREBP1*, *DGAT1*, *AGPAT6*. Therefore, in the current study, as there was a decrease in these milk FA in both SO and FO, a reduction in expression of *FABP*3 would be expected both in SO and FO. However, a reduction in expression of *FABP*3 was only observed in FO*.* Alternatively, it is possible that downregulation of *FABP3* observed with FO may be a consequence of downregulation of *PPARGC1*, which was observed in FO but not in SO.

A reduction in expression of *INSIG1* was observed on days 21, 42 and 63 in SO, whereas no effect was observed in FO. Supplementation at 2.7% DM with 18-carbon unsaturated FA such as rapeseed oil (rich in C18:1 *cis-*9), soybean oil (rich in C18:2 *cis* n-6), and linseed oil (C18:3 n-3), has inhibited de novo synthesis FA, which reduces expression of *INSIG1*^5^. In this study, SO provided 50 g/100 g of C18:2 *cis* n-6, whereas FO was composed (g/100 g) mostly of C18:2 *cis* n-6 (16), C20:5 n-3 (16), C20:5 n-3 (5), and C22:6 n-3 (8).

In Supplementary Tables S2 and S3 it is reported that expression of *SREBF1* was downregulated with SO on days 21 and 63 whereas with FO it was downregulated only on day 63. The reason would be that the increase in supply of long-chain FA from blood to the mammary gland affects transcription regulator genes^4^. Compared to control and SO, FO increased total PUFA in blood and that would partly explain FO effects on transcription factors. The response observed in *SREBP1* in both SO and FO is not consistent with that suggested by Invernizzi et al.^29^, who indicated that when lactating cows are supplemented with a blend of SO and FO, the alteration in transcription genes is generated mainly between 7 and 21 days after start of treatment. Similarly, when dairy ewes were fed with FO for 31 days, not only were transcription regulating genes (*SCAP* and *SREBF1*) downregulated but also other lipid-related genes, such as *ACSS1*, *DGAT1*, *GPAM*, and *LPIN1*^22^.

It has been described that *PPARGC1*, one of the regulatory factors of transcription, has a key role in energy, protein, glucose, and lipid metabolism, and in the latter case, *PPARGC1* regulates synthesis of fat in the mammary gland^30^. In this study, there was no variation in expression of *PPARGC1* with SO, but with FO there was downregulation from day 21 until the end of the study. As described by Sun et al.^28^ *PPARG* is responsible for regulation of expression of *DGAT1*, so it was expected that downregulation of PPARG would have generated a similar response from *DGAT1*. However, in the case of SO, despite no changes in *PPARGC1* expression, downregulation of *DGAT1* was observed at days 21 and 63, whereas in FO, *PPARGC1* was downregulated but *DGAT1* remained stable throughout the study. The explanation for this could be that *DGAT1* is also regulated by concentrations of butyrate and beta-hydroxybutyrate (BHB), because an increase of both components, or supplementation of these, generates an upregulation of *DGAT1*^28,31^. Sun et al.^28^, indicated that addition of butyrate and propionate in cell cultures of goat mammary gland epithelial cells generated upregulation of *DGAT1*, but acetate had no effect on *DGAT1* regulation. In the present study the FO group had a sustained increase in blood BHB from 0.28 to 0.69 mM between days 21 and 63^3^ whereas in milk, compared with control and SO, FO had higher contents of C4:0 (2.27 and 2.22 vs. 2.34)^1^.

### Long-term effects on relative abundance of lipid-related genes in MSC

In the control group, it was observed that on day 42, 9 genes were upregulated and their biological functions are: entry of FA into the cell (*VLDLR*, *LPL*), synthesis of FA (*FASN*), transport of FA (*FATP*), activation and transport of FA (*ACSL1*, *FABP3*), and regulation of transcription (*SCAP*, *SREBF1*, *THRSP*). As reported by Bionaz and Loor^18^ and Lee et al.^32^, expression of genes related to metabolism of FA in the mammary gland of cows and yaks changes through the lactation period. Therefore, in later lactation there is less activity or expression of these genes, and only *DGAT2* and *THRSP* show increases in expression at day 240 of lactation. Therefore, in this study, considering that on day 63 cows on average had 260 ± 35 days in lactation, a downward regulation in most of the analyzed genes would have been expected.

An important goal in this study was to determine if supplementation with FA generated a response or pattern in expression of genes over prolonged periods of lipid supplementation. Of all 20 target genes studied, only *FASD2* had the same pattern throughout the 63 days in the 3 groups of cows, without changes in its expression. The lack of change in expression of *FASD2* was unexpected. However, as discussed earlier, other studies in cows^23^ and humans^24,25^ have suggested that expression of FASD genes seems to depend not only on diet but also genetics.

In both treated groups, long-term (Table 2; Supplementary Tables S2 and S3) expression of lipid-genes remained stable or there was downregulation of them, except for *DGAT2* and *FABP4* which had increased expression at days 42 or 63. Both SO and FO, on day 63, generated a reduction in expression of *SREBF1* (transcription factor). Expression of *SREBF1* has been reported^3,5^ to be decreased by supplementation with PUFA, with concomitant downregulation in expression of lipogenic genes such as *FASN* and *LPL*.

## Limitations

Some factors need to be considered when interpreting the gene expression data from this study. For some genes, lack of significant effects on gene transcription in MSC may be related to the limited number of animals used for each treatment. Interpretations of gene expression data are limited by the absence of functional data, such as blood metabolic markers. The relative abundance software tool (REST) used in this study only allowed analysis of a reference condition (control diet) against one of the lipid-supplemented diets and therefore, future studies should analyze concurrently the effects of all diets. We chose SO and FO as supplements because they had shown effects on plasma FA, milk FA and gene expression in short-term studies. These oils contain contrasting mixtures of FA differing in chain length, number of double bonds, and bond position and configuration. To isolate some of these factors, future studies could consider using pure FA or less complex mixtures.

Overall, this study is one of the first that supplements SO and FO on a relatively long-term. Thus, the lipid-related gene responses observed provide new insights on lipid metabolism as it seems that there will be an interplay between physiological stage (days in milk) and type of lipid supplement. Amount of lipid supplement might also be important. Our approach was not to induce MFD, which has been induced or observed in other experiments^22^ and probably that is one of the reasons why we observed mild nutrigenomic effects with more prominent effects with FO.

## Conclusions

Compared to a control diet (no lipid supplement), SO downregulated *ACACA*, *INSIG1*, *DGAT1* and, *LPIN1*, while FO downregulated *ACACA*, *PPARGC1*, *LPIN1* and *FABP3* on day 63. Results suggest that FO has stronger antilipogenic effects than SO during long-term dietary oil supplementation at 2.9% of diet DM.

## Materials and methods

### Animals and experimental diets

Animal care, welfare and procedures were carried out according to the guidelines of the Animal Care Committee of the Pontificia Universidad Católica de Chile under the approved ID project 160809002. The study was conducted at the Estación Experimental Pirque of the Pontificia Universidad Católica de Chile. Fifteen pregnant cows averaging 198 ± 35 days in milk at the beginning of the study were assigned to three treatment groups based on body condition score (BCS) and milk yield. Before commencing the study, average BCS for the 3 groups was 2.8 ± 0.3, 2.6 ± 0.2, and 2.7 ± 0.3. Milk yield for the 3 groups averaged 40 ± 6, 40 ± 9, and 40 ± 8 kg/d. Details of diets and management are presented in a companion paper^1^. For 63 days all cows received a basal diet with 63% forage and 37% concentrate as a total mixed ration (Table 5). The control or basal diet contained no added lipid (n = 5 cows); treatment diets were supplemented with SO (n = 5 cows; unrefined soybean oil; 2.9% of diet DM) or FO (n = 5 cows; fish oil manufactured from salmon oil; 2.9% of diet DM). Oils were mixed manually into the daily ration for each cow. Oils were not rumen protected and were not supplied with antioxidants.

**Table 5:** Ingredients of control, soybean oil (SO), and fish oil (FO) dietary treatments.

	Diet		
	Control	SO	FO
**Ingredient composition (% DM)**			
Corn silage	32.0	31.1	31.1
Fresh alfalfa	24.0	23.3	23.3
Malt distillers	19.2	18.6	18.6
Corn grain	7.6	7.4	7.4
Canola meal	6.2	6.0	6.0
Alfalfa hay	5.0	4.9	4.9
Soybean grain	4.0	3.9	3.9
Wheat bran	1.6	1.6	1.6
Soybean oil	0	2.9	0
Fish oil	0	0	2.9
Vitamin and mineral premix^a^	0.4	0.4	0.4
**Fatty acid composition (g/100 g of FA)**			
C6:0	0.93	0.1	0.1
C10:0	0.25	nd	nd
C12:0	1.13	0.2	0.1
C14:0	10.4	0.6	7.05
C15:0	5.44	nd	4.06
C16:0	6.72	13.8	16.1
C16:1 *cis*-9	nd	1.7	4.53
C17:0	1.29	0.97	1.05
C18:0	22.5	5.17	8.72
C18:1 *cis*-9	0.92	17.9	7.94
C18:2 *cis* n-6	33.7	49.9	16.1
C18:3 n-6	7.72	2.83	2.63
C18:3 n-3	8.97	6.81	3.25
C20:5n-3	nd	nd	15.6
C22:5n-3	nd	nd	4.79
C22:6n-3	nd	nd	7.95

*nd* not detected.

^a^Contained per kg: 25 g of P; 80 g of Ca; 25 g of Mg; 1.6 g of S; 300 000 IU of vitamin A; 50 000 IU of vitamin D_3_ and 1 600 IU of vitamin E.

### Plasma samples and fatty acid analysis

At the beginning of the study (day 0) and on days 21, 42 and 63, blood samples (10 mL/cow) were obtained at 10:00 h (2 h after feeding) via jugular puncture, using tubes containing lithium heparin (BD Vacutainer; Franklin Lakes NJ, USA) and immediately centrifuged for 15 min at 3,000 g (C-28A; BOECO, Hamburg, Germany) to harvest plasma. Samples were stored at -80 °C until analyzed for FA profiles. Lipid extraction and methylation of plasma samples were done as reported previously^12^. A gas chromatograph (GC-2010) system (Shimadzu Scientific Instruments AOC-20 s, Columbia, MD, USA) equipped with a 100-m column (Rt-2560 column 100 m × 0.32 mm × 0.20 μm column; Restek, Bellefonte, PA, USA) was used. All GC conditions, FA methyl ester and reference standard were done following protocols previously reported^13^.

### Milk somatic cell sampling

Sampling for MSC followed protocols reported previously^6^. Approximately 150 mL of milk per quarter was collected from each cow four hours after routine morning milking (08:00 h) at the beginning of the study (day 0) and on days 21, 42 and 63. Udder cleaning was performed with special care: first, udders and teats were cleaned with water and soap; then, they were disinfected with chlorhexidine-based soap; lastly, teats were cleaned with RNAseZap (Ambion, Austin, TX, USA). Sterile gauze was used to cover the collection container during milk sampling. Milk was transferred from the collecting container to RNAse-free 50 mL tubes after collection.

### RNA extraction and reverse transcription

For RNA extraction, 50 mL of milk was used from each cow at each sampling period. The pellet of MSC was obtained as described by Wickramasinghe et al.^10^ and Suarez-Vega et al.^11^ with modifications reported by Vargas-Bello-Pérez et al.^3^. The RNA was extracted using QIAzol Lysis Reagent (Qiagen Inc., Valencia, CA) according to the manufacturer’s protocols. RNA quality and quantity were assessed by 1% agarose gel electrophoresis (RIN ≥ 7) and RNA quantification was measured fluorometrically using the Qubit RNA HS Assay Kit in the Qubit Fluorometer 3.0 (Invitrogen Co., Carlsbad, CA, USA). Samples were treated with RQ1 RNase-Free DNase (Cat. No. M6101; Promega, Madison, USA) to avoid genomic DNA amplification, and the absence of genomic DNA was confirmed by polymerase chain reaction (PCR) on the treated RNA^6^.

The first-strand cDNA synthesis was run on a SureCycler 8800 Thermal Cycler (Agilent Technologies Inc., Santa Clara, CA, USA) and performed using the ImProm-II Reverse Transcription System (Promega, Wisconsin, USA). Total RNA was combined with 0.5 µg/reaction oligo (dT)15 primer (Cat. No. C1101; Promega, Madison, USA) to a final volume of 5 µL and was incubated at 70 °C for 5 min. Next, 15 µL of transcription mix (4.6 µL of ImProm-II 5 × Reaction Buffer, 2.25 mM of MgCl2, 0.5 mM each of dNTP and Recombinant RNasin Ribonuclease Inhibitor (Promega, Cat. No. N2511, Madison, USA), in the amount of 0.5 µL, and 1 µL ImProm-II Reverse Transcriptase (Promega, Cat. No. A3802, Madison, USA) was added. Following addition of transcription mix, the reaction was maintained at 25 °C for 5 min and was then transferred to 42 °C for 60 min. Reverse transcription reactions were stopped by heating the mixture at 70 °C for 15 min. cDNA was stored at − 80 °C until use^6^.

### Gene abundance

Genes and primer-pairs used in the current study have been reported previously^6^ and the quantitative PCR performance for all genes including internal controls is shown in Supplementary Table S1. The target genes are related to FA import into cells (*LPL*, *VLDLR*), FA synthesis and desaturation (*ACACA*, *FADS2*, *FASN*, *SCD*), acetate and FA activation and intra-cellular transport (*ACSL1*, *ACSS2*, *FABP3*, *FABP4*, *FATP*), triacylglycerol synthesis (*DGAT1*, *DGAT2*, *LPIN1*), lipid droplet formation (*ADFP*) and regulation of transcription (*INSIG1*, *PPARGC1*, *SCAP*, *SREBF1*, *THRSP*). For normalization of cDNA loading, all samples were run in parallel using the following housekeeping genes: *GAPDH* (glyceraldehyde 3-phosphate dehydrogenase), *EIF3K* (eukaryotic translation initiation factor 3 subunit K) and *UXT* (ubiquitously expressed prefoldin like chaperone). Relative mRNA abundance levels of the target genes and the housekeeping genes were quantified using real-time PCR analysis with AriaMx (Agilent Technologies) and details on the amplification of specific PCR products are reported in our companion paper^3^. The PCR primer efficiency (E) was calculated for each gene fluorescence curve with LinRegPCR 12.18 software. Relative abundance of genes involved in lipid metabolism in MSC from cows fed control on days 42 and 63 was compared with relative abundance of day 21 to evaluate fold-change^6^.

### Statistical analysis

A model including diet, time, and diet × time as fixed effects and cow within treatment as a random effect was used to examine differences in plasma FA profiles. All data were analyzed using the MIXED procedure in SAS (SAS Institute Inc., Cary, NC). Least squares mean were separated using the PDIFF (Piecewise Differentiable) statement in SAS. The relative abundance software tool (REST) was used to analyze qPCR results. This software incorporates PCR efficiency correction and reference gene normalization. It integrates a statistical analysis randomization algorithm to calculate the statistical difference of variation between two groups and a bootstrapping technique, which provides 95% confidence interval for abundance ratios^33^. To test the effect of diet, data on relative gene abundance were based on comparing control vs. SO and control vs. FO at each sampling time (21, 42, and 63 days). To test the long-term effects of lipid supplementation, data on relative gene abundance were based on comparisons between day 21 vs. day 42, and day 21 vs. day 63^6^. Relative quantification of gene abundance and the statistical analysis were performed with the REST software.

## Supplementary information

Supplementary Information.
