# Supplementary material for: Effects of dietary polyunsaturated fatty acid sources on expression of lipid-related genes in bovine milk somatic cells
Source: Sci Rep. 2020 Sep 9;10:14850. doi: 10.1038/s41598-020-71930-x (PMC7481231; doi:10.1038/s41598-020-71930-x)
Supplement: Supplementary file 1 — Supplementary Information. [file 41598_2020_71930_MOESM1_ESM.docx]

**Effects of dietary polyunsaturated fatty acid sources on expression of lipid-related genes in bovine milk somatic cells**

Einar Vargas-Bello-Pérez^*1, 2^, Nathaly Cancino-Padilla^2^, Carolina Geldsetzer-Mendoza^2^, María Sol Morales^3^, Heidi Leskinen^4^, Philip C. Garnsworthy^5^, Juan J. Loor^6^, Jaime Romero^7^

^1^University of Copenhagen, Faculty of Health and Medical Sciences, Department of Veterinary and Animal Sciences, Grønnegårdsvej 3, DK-1870 Frederiksberg C, Denmark.

^2^Departamento de Ciencias Animales, Facultad de Agronomía e Ingeniería Forestal, Pontificia Universidad Católica de Chile, Santiago, Chile. Casilla 306. C.P. 6904411.

^3^Departamento de Fomento de la Producción Animal, Facultad de Ciencias Veterinarias y Pecuarias. Universidad de Chile. Av. Santa Rosa 11735, La Pintana, Santiago, Chile.

^4^Milk Production, Production Systems, Natural Resources Institute Finland (Luke), Jokioinen, FI-31600, Finland

^5^The University of Nottingham, School of Biosciences, Sutton Bonington Campus, Loughborough, LE12 5RD, United Kingdom.

^6^University of Illinois, Mammalian NutriPhysioGenomics, Department of Animal Sciences and Division of Nutritional Sciences, Urbana 61801, United States.

^7^Laboratorio de Biotecnología en Alimentos, Unidad de Alimentos, Instituto de Nutrición y Tecnología de los Alimentos, Universidad de Chile, Avda. El Líbano 5524, Macul, Santiago, 7830490, Chile

*Correspondence: Einar Vargas-Bello-Pérez. University of Copenhagen, Faculty of Health and Medical Sciences, Department of Veterinary and Animal Sciences, Telephone: +45 35 32 60 98. E-mail: [evargasb@sund.ku.dk](mailto:evargasb@sund.ku.dk)

**Supplementary material**

Table S1. Gene, name, GenBank accession number, and qPCR performance of target genes and internal controls (*GAPDH*, *EIF3K* and *UXT*)

| Gene | Name | Accession # | Median Ct^1^ | Median ΔCt^2^ | Slope | (R2)^3^ | Efficiency^4^ |
| --- | --- | --- | --- | --- | --- | --- | --- |
| *ACACA* | Acetyl-coenzyme A carboxylase alpha | AJ132890 | 26.16 | 3.82 | -3.33 | 0.997 | 1.381 |
| *FADS2* | Fatty acid desaturase 2 (delta-6 desaturase) | NM_001083444 | 27.40 | 5.31 | -3.38 | 0.997 | 0.741 |
| *FASN* | Fatty acid synthase | NM_001012669 | 21.95 | 0.53 | -3.11 | 0.937 | 132.817 |
| *SCD* | Stearoyl-CoA desaturase (delta-9-desaturase) | AY241933 | 23.96 | 0.51 | -3.51 | 0.996 | 9.593 |
| *ADFP* | Adipose differentiation related protein | BC102211 | 19.28 | -2.38 | -3.47 | 0.995 | 10.308 |
| *INSIG1* | Insulin induced gene 2 | NM_001077909.1 | 25.89 | 4.29 | -3.38 | 0.994 | 0.82 |
| *SCAP* | SREBP cleavage activating protein | NM_00101889 | 25.86 | 3.54 | -3.29 | 0.962 | 4.225 |
| *SREBF1* | Sterol regulatory element-binding transcription factor 1 | NM_001113302 | 23.64 | 1.70 | -3.51 | 0.999 | 1.859 |
| *THRSP* | Thyroid hormone responsive SPOT14 | AY656814 | 29.49 | 7.81 | -3.77 | 0.998 | 3.888 |
| *PPARGC1* | PPAR gamma, coactivator 1 alpha | NM_177945 | 28.57 | 6.74 | -3.33 | 0.988 | 0.883 |
| *DGAT1* | Diacylglycerol acyltransferase 1 | NM_174693 | 24.58 | 2.61 | -3.54 | 0.995 | 0.572 |
| *DGAT2* | Diacylglycerol acyltransferase 2 | BT030532.1 | 22.94 | 1.30 | -3.38 | 0.996 | 1.406 |
| *LPIN1* | Lipin 1 | NM_001206156 | 25.31 | 3.77 | -3.57 | 0.995 | 0.598 |
| *LPL* | Lipoprotein lipase | BC118091 | 20.76 | -1.16 | -3.43 | 0.997 | 1.145 |
| *FATP* | Long-chain fatty acid transport protein | NM_001033625 | 28.63 | 6.21 | -3.55 | 0.998 | 2.045 |
| *VLDLR* | Very-Low Density Lipoprotein Receptor | AJ609502 | 25.19 | 2.97 | -3.62 | 0.997 | 0.96 |
| *ACSL1* | Acyl-CoA synthetase long-chain family member 1 | BC119914 | 22.68 | 0.86 | -3.42 | 0.996 | 1.106 |
| *ACSS2* | Acyl-CoA synthetase short-chain family member 2 | BC134532 | 21.72 | 0.30 | -3.32 | 0.996 | 1.255 |
| *FABP3* | Fatty acid-binding protein 2 | NM_174313 | 19.97 | -2.16 | -3.45 | 0.995 | 3.918 |
| *FABP4* | Fatty acid-binding protein 4 | NM_174314 | 20.01 | -1.86 | -3.39 | 0.996 | 0.514 |
| *GAPDH* | Glyceraldehyde 3-phosphate dehydrogenase | NM_001034034 | 19.59 | - | -3.63 | 0.998 | 1.511 |
| *EIF3K* | Eukaryotic translation initiation factor 3 subunit K | NM_001034489 | 22.70 | - | -3.41 | 0.994 | 0.915 |
| *UXT* | Ubiquitously expressed prefoldin like chaperone | NM_001037471 | 24.40 | - | -3.41 | 0.995 | 0.723 |

^1^The median is calculated considering all samples.

^2^The median of ΔCt is calculated as [Ct gene-geometric mean of Ct internal controls] for each sample.

^3^R2 represents the coefficient of determination of the standard curve.

^4^Efficiency was calculated as [-10^(-1/Slope)^].

Table S2. Relative expression of genes involved in lipid metabolism in milk somatic cells from cows fed with soybean oil on days 21, 42 and 63 using the relative abundance of the onset of the experiment (no fat supplementation) as the reference condition

| Gene | Day | Relative abundance | Standard error | *P*-value | Regulation |
| --- | --- | --- | --- | --- | --- |
| *ACACA* | 21 | 1.466 | 0.301 - 8.665 | 0.355 |  |
|  | 42 | 0.09 | 0.016 - 0.524 | <0.001 | DOWN |
|  | 63 | 0.129 | 0.026 - 0.737 | <0.001 | DOWN |
| *FADS2* | 21 | 1.1 | 0.529 - 2.166 | 0.583 |  |
|  | 42 | 1.048 | 0.533 - 2.109 | 0.804 |  |
|  | 63 | 1.259 | 0.687 - 2.355 | 0.150 |  |
| *FASN* | 21 | 0.834 | 0.065 - 10.581 | 0.778 |  |
|  | 42 | 0.735 | 0.131 - 3.297 | 0.514 |  |
|  | 63 | 0.749 | 0.155 - 5.111 | 0.518 |  |
| *SCD* | 21 | 0.741 | 0.138 - 4.858 | 0.496 |  |
|  | 42 | 0.714 | 0.093 - 4.062 | 0.487 |  |
|  | 63 | 0.099 | 0.006 - 2.876 | 0.004 | DOWN |
| *ADFP* | 21 | 0.936 | 0.642 - 1.380 | 0.555 |  |
|  | 42 | 0.894 | 0.463 - 2.560 | 0.591 |  |
|  | 63 | 0.997 | 0.511 - 2.281 | 0.983 |  |
| *INSIG1* | 21 | 0.363 | 0.123 - 1.282 | 0.004 |  |
|  | 42 | 0.413 | 0.146 - 1.155 | 0.001 | DOWN |
|  | 63 | 0.201 | 0.081 - 0.521 | <0.001 | DOWN |
| *SCAP* | 21 | 0.68 | 0.201 - 1.883 | 0.217 |  |
|  | 42 | 0.43 | 0.091 - 1.696 | 0.037 | DOWN |
|  | 63 | 0.691 | 0.223 - 2.042 | 0.231 |  |
| *SREBF1* | 21 | 0.408 | 0.237 - 0.697 | <0.001 | DOWN |
|  | 42 | 0.822 | 0.501 - 1.402 | 0.169 |  |
|  | 63 | 0.564 | 0.376 - 0.876 | <0.001 | DOWN |
| *THRSP* | 21 | 0.587 | 0.123 - 2.603 | 0.161 |  |
|  | 42 | 0.187 | 0.069 - 0.488 | <0.001 | DOWN |
|  | 63 | 0.508 | 0.131 - 2.787 | 0.084 |  |
| *PPARGC1* | 21 | 0.668 | 0.054 - 7.272 | 0.466 |  |
|  | 42 | 0.534 | 0.151 - 2.018 | 0.084 |  |
|  | 63 | 0.843 | 0.103 - 5.713 | 0.696 |  |
| *DGAT1* | 21 | 0.403 | 0.219 - 0.713 | <0.001 | DOWN |
|  | 42 | 0.749 | 0.379 - 1.752 | 0.167 |  |
|  | 63 | 0.542 | 0.329 - 0.909 | 0.001 | DOWN |
| *DGAT2* | 21 | 0.535 | 0.121 - 1.890 | 0.100 |  |
|  | 42 | 2.048 | 0.653 - 7.041 | 0.038 | UP |
|  | 63 | 2.108 | 0.706 - 5.874 | 0.018 | UP |
| *LPIN1* | 21 | 0.544 | 0.106 - 2.757 | 0.120 |  |
|  | 42 | 0.53 | 0.170 - 1.576 | 0.035 | DOWN |
|  | 63 | 0.738 | 0.200 - 2.893 | 0.384 |  |
| *LPL* | 21 | 1.233 | 0.602 - 2.572 | 0.294 |  |
|  | 42 | 0.753 | 0.282 - 1.747 | 0.231 |  |
|  | 63 | 1.022 | 0.415 - 2.405 | 0.922 |  |
| *FATP* | 21 | 0.777 | 0.371 - 1.696 | 0.210 |  |
|  | 42 | 1.591 | 0.761 - 3.338 | 0.013 | UP |
|  | 63 | 0.56 | 0.265 - 1.137 | 0.003 | DOWN |
| *VLDLR* | 21 | 1.014 | 0.568 - 1.826 | 0.940 |  |
|  | 42 | 1.011 | 0.523 - 1.916 | 0.949 |  |
|  | 63 | 1.032 | 0.566 - 1.958 | 0.860 |  |
| *ACSL1* | 21 | 1.079 | 0.501 - 2.504 | 0.699 |  |
|  | 42 | 0.493 | 0.183 - 1.357 | 0.012 | DOWN |
|  | 63 | 1.144 | 0.549 - 2.708 | 0.524 |  |
| *ACCS2* | 21 | 0.876 | 0.182 - 4.144 | 0.706 |  |
|  | 42 | 0.518 | 0.179 - 1.852 | 0.046 | DOWN |
|  | 63 | 0.613 | 0.146 - 2.800 | 0.206 |  |
| *FABP3* | 21 | 0.638 | 0.074 - 4.830 | 0.390 |  |
|  | 42 | 0.463 | 0.065 - 3.934 | 0.152 |  |
|  | 63 | 0.569 | 0.068 - 5.070 | 0.283 |  |
| *FABP4* | 21 | 0.806 | 0.123 - 6.379 | 0.669 |  |
|  | 42 | 0.537 | 0.080 - 8.220 | 0.334 |  |
|  | 63 | 1.491 | 0.191 - 11.720 | 0.397 |  |

Table S3. Relative expression of genes involved in lipid metabolism in milk somatic cells from cows fed with fish oil on days 21, 42 and 63 using the relative abundance of the onset of the experiment (no fat supplementation) as the reference condition

| Gene | Day | Relative abundance | Standard error | *P*-value | Regulation |
| --- | --- | --- | --- | --- | --- |
| *ACACA* | 21 | 0.086 | 0.021 - 0.430 | <0.001 | DOWN |
|  | 42 | 0.055 | 0.011 - 0.390 | <0.001 | DOWN |
|  | 63 | 0.068 | 0.011 - 0.351 | <0.001 | DOWN |
| *FADS2* | 21 | 0.686 | 0.268 - 2.044 | 0.124 |  |
|  | 42 | 0.652 | 0.221 - 1.943 | 0.155 |  |
|  | 63 | 0.965 | 0.334 - 2.866 | 0.897 |  |
| *FASN* | 21 | 0.445 | 0.045 - 3.267 | 0.095 |  |
|  | 42 | 0.259 | 0.014 - 4.629 | 0.030 | DOWN |
|  | 63 | 0.205 | 0.022 - 2.465 | 0.004 | DOWN |
| *SCD* | 21 | 0.499 | 0.086 - 2.302 | 0.086 |  |
|  | 42 | 0.122 | 0.008 - 2.239 | 0.004 | DOWN |
|  | 63 | 0.014 | 0.002 - 0.108 | <0.001 | DOWN |
| *ADFP* | 21 | 0.931 | 0.338 - 2.126 | 0.748 |  |
|  | 42 | 0.462 | 0.037 - 2.179 | 0.129 |  |
|  | 63 | 1.33 | 0.552 - 2.917 | 0.228 |  |
| *INSIG1* | 21 | 1.494 | 0.387 - 11.075 | 0.329 |  |
|  | 42 | 0.851 | 0.154 - 5.180 | 0.730 |  |
|  | 63 | 0.799 | 0.184 - 5.502 | 0.605 |  |
| *SCAP* | 21 | 1.18 | 0.576 - 2.273 | 0.329 |  |
|  | 42 | 0.279 | 0.034 - 1.113 | 0.005 | DOWN |
|  | 63 | 0.371 | 0.196 - 0.679 | <0.001 | DOWN |
| *SREBF1* | 21 | 1.064 | 0.492 - 2.151 | 0.747 |  |
|  | 42 | 1.41 | 0.479 - 11.380 | 0.346 |  |
|  | 63 | 0.726 | 0.408 - 1.305 | 0.044 | DOWN |
| *THRSP* | 21 | 0.298 | 0.069 - 1.829 | 0.010 | DOWN |
|  | 42 | 0.449 | 0.030 - 13.938 | 0.319 |  |
|  | 63 | 0.433 | 0.083 - 2.864 | 0.087 |  |
| *PPARGC1* | 21 | 0.314 | 0.051 - 1.718 | 0.009 | DOWN |
|  | 42 | 0.262 | 0.029 - 2.259 | 0.015 | DOWN |
|  | 63 | 0.221 | 0.034 - 1.643 | 0.004 | DOWN |
| *DGAT1* | 21 | 1.28 | 0.736 - 2.150 | 0.117 |  |
|  | 42 | 2.089 | 0.725 - 27.553 | 0.105 |  |
|  | 63 | 1.279 | 0.798 - 1.960 | 0.073 |  |
| *DGAT2* | 21 | 1.393 | 0.296 - 4.775 | 0.347 |  |
|  | 42 | 4.636 | 0.877 - 34.487 | 0.004 | UP |
|  | 63 | 1.227 | 0.482 - 4.120 | 0.476 |  |
| *LPIN1* | 21 | 0.345 | 0.061 - 1.375 | 0.009 | DOWN |
|  | 42 | 0.277 | 0.043 - 1.691 | 0.005 | DOWN |
|  | 63 | 0.277 | 0.058 - 1.095 | 0.001 | DOWN |
| *LPL* | 21 | 0.849 | 0.307 - 2.641 | 0.572 |  |
|  | 42 | 0.399 | 0.073 - 1.659 | 0.050 | DOWN |
|  | 63 | 0.941 | 0.346 - 2.879 | 0.838 |  |
| *FATP* | 21 | 2.37 | 1.528 - 3.622 | <0.001 | UP |
|  | 42 | 3.795 | 1.008 - 83.973 | <0.001 | UP |
|  | 63 | 0.696 | 0.379 - 1.347 | 0.109 |  |
| *VLDLR* | 21 | 0.722 | 0.444 - 1.153 | 0.013 | DOWN |
|  | 42 | 2.253 | 0.716 - 34.531 | 0.068 |  |
|  | 63 | 0.903 | 0.511 - 1.663 | 0.497 |  |
| *ACSL1* | 21 | 0.551 | 0.214 - 1.396 | 0.016 | DOWN |
|  | 42 | 1.542 | 0.346 - 12.610 | 0.327 |  |
|  | 63 | 0.999 | 0.429 - 2.000 | 0.996 |  |
| *ACCS2* | 21 | 0.265 | 0.062 - 1.175 | 0.002 | DOWN |
|  | 42 | 0.139 | 0.022 - 1.382 | 0.001 | DOWN |
|  | 63 | 0.255 | 0.055 - 1.610 | 0.002 | DOWN |
| *FABP3* | 21 | 0.237 | 0.042 - 1.389 | 0.002 | DOWN |
|  | 42 | 0.122 | 0.013 - 1.645 | 0.002 | DOWN |
|  | 63 | 0.21 | 0.048 - 1.614 | 0.001 | DOWN |
| *FABP4* | 21 | 1.181 | 0.240 - 5.472 | 0.665 |  |
|  | 42 | 4.042 | 0.870 - 20.656 | 0.001 | UP |
|  | 63 | 2.476 | 0.938 - 6.783 | <0.001 | UP |
